# Evaluation of aversive behavior in *Rattus norvegicus* experimentally infected by two distinct strains of *Toxoplasma gondii* (ME49 and VEG): study of epigenetic markers

**DOI:** 10.1590/0037-8682-0122-2022

**Published:** 2022-10-24

**Authors:** Sergio Vieira dos Santos, Giulio de los Santos Fortuna, Lariane Monteiro Barbosa, Luciana Regina Meireles, Érico Silva Tiago, Pedro Paulo Chieffi

**Affiliations:** 1 Santa Casa de São Paulo, Faculdade de Ciências Médicas, Departamento de Ciências Patológicas, Laboratório de Parasitologia, São Paulo, SP, Brasil.; 2 Universidade de São Paulo, Faculdade de Medicina, Instituto de Medicina Tropical, Laboratório de Protozoologia, São Paulo, SP, Brasil.

**Keywords:** Toxoplasma gondii, ME49 and VEG strains, Rattus norvegicus, Behavior alterations, Arginine-vasopressin promoter gene

## Abstract

**Background:**

Behavioral changes in *Rattus norvegicus* infected with two strains of *Toxoplasma gondii* (ME49 and VEG) were investigated.

**Methods:**

Rats were evaluated for motor activity and aversion or attraction to cat urine 60 days after infection. After euthanasia, arginine-vasopressin gene methylation in the central nervous system was evaluated.

**Results:**

A significant difference was observed in the methylation of the arginine-vasopressin promoter gene between rats infected with the ME49 and VEG strains.

**Conclusions:**

Although differences were not observed in many parameters, significant differences were observed in the methylation of the arginine-vasopressin promoter gene in rats infected with the two studied strains.

Epigenetic modifications in host cells in response to infection by intracellular pathogens are a relatively unexplored field. Knowledge of the mechanisms that lead to behavioral modifications, whether epigenetic, neuromodulatory, or simply an infection side effect, is important for the creation of preventive measures and the treatment for parasitic infections associated with behavioral changes^1^.

*Toxoplasma gondii* is an obligate intracellular parasite capable of infecting birds and mammals, including humans. Although *T. gondii* infections are mostly asymptomatic in humans, significant morbidity can occur in immunocompromised patients^2^ ^,^ ^3^. Congenital infection is a concern during the prenatal period because of its possible severity^4^.

In rodents, experimental *T. gondii* infection can generate impaired memory and learning, as well as attenuate the innate aversion of mice to predator urine, making it an attractant. These changes are believed to benefit the protozoan by facilitating its transmission to felids, the definitive host^5^. Dass and Vyas^6^ found that the medial post-dorsal (MePD) region of the amygdala is activated in rats infected with *T*. *gondii* and exposed to the odor of cat urine, which is an area associated with reproductive and social behavior. Using epigenetic tools, the same authors verified that the group infected with *T. gondii* showed hypomethylation in the arginine-vasopressin promoter (AVP) gene site in the MePD region. 

In the present study, we investigated the relationship between infections caused by distinct strains of *T*. *gondii* (ME49 and VEG), behavioral changes (memory, learning, and aversion to cat urine), and methylation of the AVP gene in the amygdala region of the brains of infected *Rattus norvegicus*.

Thirty male rats (6-8 weeks of age) were divided into the following three groups of 10 animals each: G1, rats infected with *T. gondii* ME49 strain; G2, rats infected with *T. gondii* VEG strain; G3, non-infected control rats. Rats in groups 1 and 2 were infected orally with 10 *T. gondii* cysts from each strain. 

After 2 months of infection, a T-maze experiment, as used by Bezerra et al.^1^, was conducted to analyze aversive behavior to the odor of cat urine. The animals were habituated to the apparatus for three consecutive days for 300 seconds. On the fourth day, each rodent was tested in the apparatus, with or without predator urine. Each arm of the apparatus had a towel containing 2 mL of cat urine or a towel without urine. Each animal had 300 seconds to complete the test, during which the frequency of entry into and dwelling time in the arms with and without cat urine were analyzed. Other behavioral experiments included the open field test where the immobility time, time spent in the periphery, in the center, between the center and the periphery, grooming time, number of quadrants in which the rat moved, number of rearings, and amount of excreted feces were evaluated. The movement of the animals was also evaluated using an actometer (Ugo Basile 47420 Multiple Activity Cage) which determined the frequencies of vertical and horizontal movements of the animals. The observation time for each device was 300 seconds^1^ ^,^ ^7^. 

After behavioral evaluation, the animals were anesthetized (ketamine and xylazine, 10/0.5 mg per 100 g body weight via intraperitoneal injection) and euthanized by exsanguination to remove the central nervous system and analyze the methylation of the AVP gene in five animals from each group. The section containing the amygdala region was obtained using a 2 mm Rat Brain Cutting Array. Microdissection of the region was performed using an autoclaved Pasteur pipette to obtain microfragments, which were transferred to a 2.0 mL microtube with 250 µL of lysis buffer and stored at −80°C for subsequent DNA extraction. 

For genomic extraction, a kit (DNeasy Blood & Tissue) from QIAGEN was used. The concentration of DNA extracted from each microfragment was determined using the Thermo Scientific^TM^ NanoDrop 2000. Methylation of the AVP gene was quantified by digestion with methylation-sensitive restriction enzymes (MSRE) in combination with quantitative PCR. Primers and marker sites (site 3: F-CGCCTTCAAAGCTCTAGTGG, R-TATGAACCCCAAGGGAAGTC; site 4: F-GGCCTTTGGCTCTATGTTC, R-TTGAGGGTCACCTGGAAATC) were adapted from Auger et al.^8^

After extraction, the DNA obtained was digested with the following MSREs: Promega HpaII (REF-R6311), which cleaves unmethylated CCGG, and BsH1236I (BstUI; Thermo Fisher Scientific^TM^, ER0921), which cleaves unmethylated CGCG. After DNA treatment with the enzymes, quantitative PCR was performed using the SYBR Green® system (Bio-Rad, cat: #172-5271) and the Applied Biosystems® 7500 Real-Time PCR System.

The Student's *t*-test was used to analyze statistical differences between the DNA treated and untreated with enzymes. Analysis of variance was used to analyze the variance in the behavioral variables. Statistical significance was set at P <0.05. 

This study was approved by the Ethics Committee on the Use of Animals in the Brotherhood of Santa Casa de São Paulo (CEUA-ISCMSP/FCMSCSP), registered under number 004/17.

In the T-maze, significant differences were not observed between the infected groups (G1 and G2) and the non-infected control group (G3) in the dwelling time or frequency of entry into the arm of the T-maze in the presence of cat urine.

In the open field test, the following statistically significant differences were observed: (i) longer dwelling time at the periphery by rats infected with the VEG strain; (ii) longer dwelling time between the center and periphery for the group infected with the ME49 strain; and (iii) lower number of quadrants in which movement occurred in rats infected with the VEG strain. (Figure 1)

**FIGURE 1:**
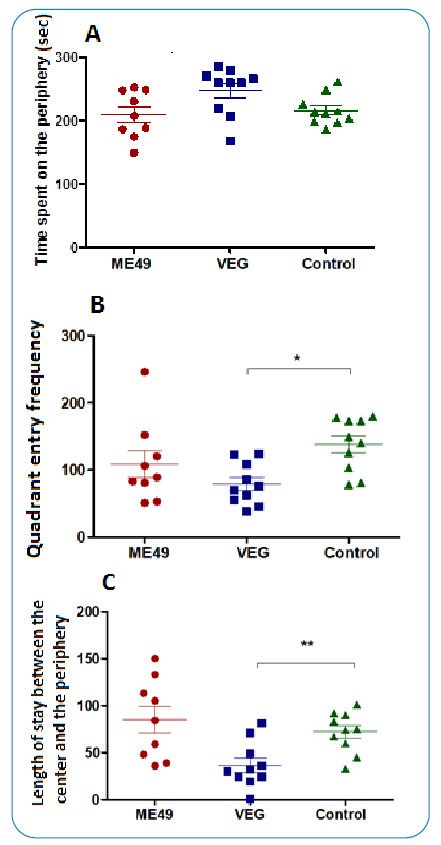
Rats infected with *T. gondii* ME49 and VEG strains and uninfected rats after 2 months of infection in the open field: **(A)** Time spent on the periphery, **(B)** number of frequented quadrants, and **(C)** time spent between the center and periphery. Statistical analyses with Kruskal-Wallis test and Dunn post test; *p =0,0168, **p =0,004; N=10.

Using an actometer, a higher frequency of vertical movements in the central column among the rats infected with the ME49 strain was detected compared with the rats in the control group (Control: 199,91; ME49: 199,1; P <0,05; confidence interval [CI], 95%; 4.488 to 169,9). 

The evaluation of AVP gene methylation in the amygdala was performed using quantitative PCR with untreated and restriction enzyme-treated (HpaII and BstUI) DNA samples and subsequent statistical analysis using the Student's *t*-test. A significantly higher cycle threshold (Ct) value was observed after treatment with the HpaII enzyme in rats in the *T. gondii* VEG strain-infected group (P <0.05). In the rats of the ME49 strain-infected and control groups, no significant changes were observed in Ct values (Figure 2). In addition, the rats infected with the VEG strain showed a higher ΔCt value (Ct value of the sample not treated with HpaII subtracted by the Ct value of the treated sample) than the control rats (P <0.05), but there was no statistically significant change in the ME49 strain-infected rats (Figure 2D). A significantly higher Ct value was obtained after treatment with the BstUI enzyme in the control group (P <0.05) than in the infected groups (Figure 3A). However, the ΔCt value was not significantly different between the infected and control groups (Figure 3D). 

**FIGURE 2:**
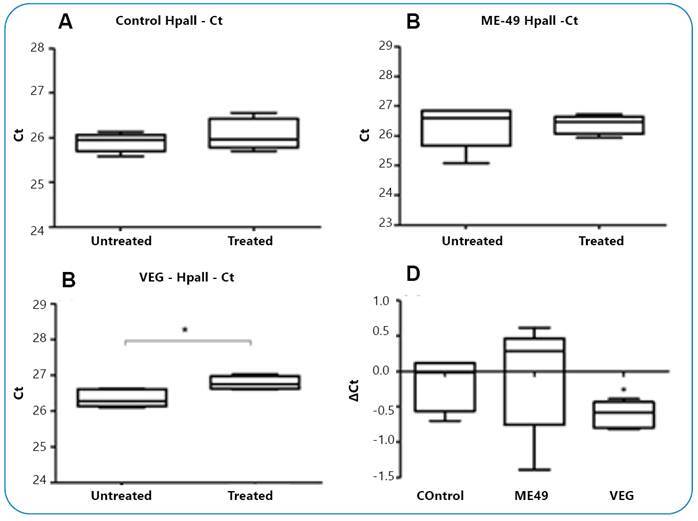
**(A)** Ct values for DNA samples not treated and treated with the HpaII enzyme in uninfected rats, **(B)** rats infected with *T. gondii* ME49 strain, and **(C)** rats infected with the VEG strain (t = 3.311; df = 4; CI, 95%; -0.8096 to -0.07117; *p =0,0296, paired Student´s *t-test*). **(D)** Represents the ΔCt value in the control and infected rat groups (t=2.340; df = 8; CI, 95%; 0.006140 to 0.8511, unpaired Student´s *t-test*).

**FIGURE 3:**
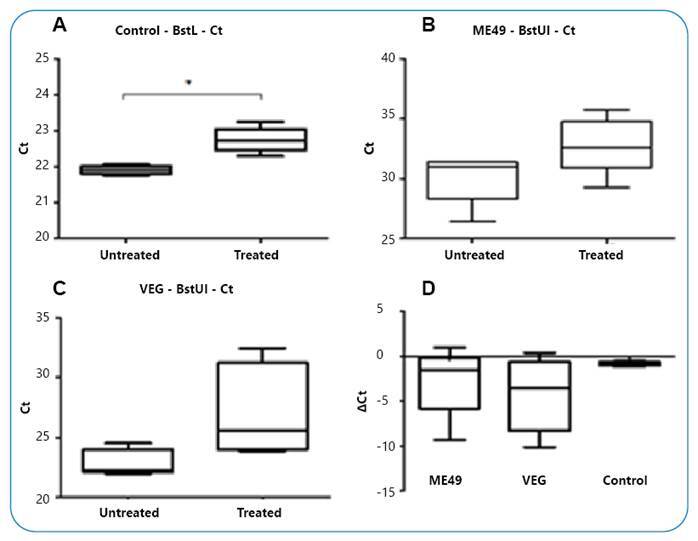
**(A)** Ct values for DNA samples not treated and treated with the BstUI enzyme in uninfected rats (t = 7.687; df = 4; CI, 95%; -1.138 to -0.5343; *p =0,0015, paired Student´s *t-test*), **(B)** rats infected with *T. gondii* ME49 strain, and **(C)** rats infected with the VEG strain. **(D)** Represents the ΔCt value in the control and infected rat groups.

Behavioral manipulation of the host by the parasite through epigenetics is an interesting mechanism that may have undergone selective pressure to improve the transmission of the infectious agent. This type of mechanism has been reported in rats infected with the protozoan *T*. *gondii*, in which a reduction in the innate aversion of the rodent to the urine odor of its predator is observed, which contributes to increasing the transmission of the protozoan to the definitive host^9^ ^-^ ^12^. The same result was not obtained regarding aversion to cat urine odor in the present study, and a statistically significant increase in dwelling time or the frequency of entry into the T-maze arm with cat urine was not observed between infected and control rats. 

This result may be explained by the specific brain regions where the cysts were located in each animal, as well as the time elapsed between infection and when the experiment was conducted, as already observed by Evans et al.^13^ Similarly, Bezerra et al.^1^ demonstrated that mice infected with the VEG strain had a higher number of entries in the Y-maze arm containing cat urine than mice infected with the ME49 strain after 6 weeks of infection.

Conversely, Queiroz et al.^7^ reported a higher frequency of head dipping in *T. gondii*-infected rats after 60 days of infection, which signals greater exploratory behavior and, therefore, greater susceptibility to predation, but not after 30 or 120 days. Furthermore, the cat urine used may have influenced the results because a greater attraction of infected rats has been reported when the urine is from a wild cat^12^ and a moderate amount of urine was used in the experiment^11^. 

Other parameters showed changes in the exploratory behavior of infected rats. When using an actometer, rats infected with the ME49 strain, but not the VEG strain, exhibited a higher frequency of vertical movements compared with the control rats, indicating greater exposure to possible predators and changes in the behavioral modulation of the rat based on different parasite strains. 

The mechanism that results in the manipulation of rodent behavior by *T. gondii* infection involves the following: (i) changes in the neural activity of limbic areas, such as the amygdala^6^ ^,^ ^11^ and dopaminergic activity in the nucleus accumbens^14^, although there is no predilection in the distribution of parasite cysts in the prosencephalon^13^; (ii) increased expression of testosterone-related genes and, consequently, increased testosterone production in male rats^6^; and (iii) hypomethylation of the arginine-vasopressin gene in the MePD region of the amygdala in male^6^ but not female rats^9^.

The present study showed that male rats infected with the *T. gondii* VEG strain had higher Ct and ΔCt values after treatment with the DNA sample obtained from the amygdala with the HpaII enzyme (P <0.05), which was not observed in rats infected with the ME49 strain and in the control rats, indicating that hypomethylation of the AVP gene regions susceptible to HpaII enzymes in the amygdala differs based on *T. gondii* strains. However, a higher Ct value after treatment of DNA samples with the BstUI enzyme was only detected in the control group (p <0.05), indicating a variation in methylation alteration of different AVP gene regions in *T. gondii*-infected rats. In addition, the data of the treatment with the BstUI enzyme showed a higher standard deviation, which may mask a possible statistically significant difference in the group infected with the VEG strain (P =0.0801 for Ct variation between treatment and no treatment with the BstUI enzyme). 

The results obtained in this study indicate that the epigenetic and behavioral changes resulting from *T. gondii* infection in rats vary based on the protozoan strain. Although hypomethylation of site 3 in the AVP region of the amygdala was detected in rats infected with the VEG strain, the same was not observed in the ME49 strain. The behavioral parameters analyzed in the experiments also varied depending on the infecting strain. Additionally, variations in the methylation changes produced in different AVP gene regions by *T. gondii* infection were detected.

Furthermore, a lack of correlation was found between the epigenetic changes detected and the behavioral manifestations of the rats, because hypomethylation of site 3 in the AVP gene region of the amygdala was detected in the rats infected with the VEG strain. However, a corresponding reduction in aversion to cat urine odor was not observed and the rats that showed an increase in exploratory behavior were infected with the ME49 strain. This may be a consequence of the time elapsed after infection with the protozoan, which could produce oscillating behavioral changes. Conversely, other environmental variables that more accurately simulate the predation environment of rats by cats, such as the movement and smell of cat fur, were not considered in behavioral experiments^15^.

In conclusion, this study indicated that epigenetic manipulation of the AVP gene in the amygdala of male rats infected with the *T. gondii* protozoan varies based on the infecting strain and the promoter region analyzed. Furthermore, genetic hypomethylation did not necessarily result in the expected attenuation of aversion to cat urine odor and a greater exploratory character in *T. gondii*-infected rats. In future studies, an analysis of temporal oscillations of behavioral manipulation in circumstances closer to environmental reality is required.
